# Evaluating Tools for Live Imaging of Structural Plasticity at the Axon Initial Segment

**DOI:** 10.3389/fncel.2016.00268

**Published:** 2016-11-23

**Authors:** Adna S. Dumitrescu, Mark D. Evans, Matthew S. Grubb

**Affiliations:** Centre for Developmental Neurobiology, King’s College LondonLondon, UK

**Keywords:** axon initial segment, plasticity, imaging, dentate granule cell

## Abstract

The axon initial segment (AIS) is a specialized neuronal compartment involved in the maintenance of axo-dendritic polarity and in the generation of action potentials. It is also a site of significant structural plasticity—manipulations of neuronal activity *in vitro* and *in vivo* can produce changes in AIS position and/or size that are associated with alterations in intrinsic excitability. However, to date all activity-dependent AIS changes have been observed in experiments carried out on fixed samples, offering only a snapshot, population-wide view of this form of plasticity. To extend these findings by following morphological changes at the AIS of individual neurons requires reliable means of labeling the structure in live preparations. Here, we assessed five different immunofluorescence-based and genetically-encoded tools for live-labeling the AIS of dentate granule cells (DGCs) in dissociated hippocampal cultures. We found that an antibody targeting the extracellular domain of neurofascin provided accurate live label of AIS structure at baseline, but could not follow rapid activity-dependent changes in AIS length. Three different fusion constructs of GFP with full-length AIS proteins also proved unsuitable: while neurofascin-186-GFP and Na_V_β4-GFP did not localize to the AIS in our experimental conditions, overexpressing 270kDa-AnkyrinG-GFP produced abnormally elongated AISs in mature neurons. In contrast, a genetically-encoded construct consisting of a voltage-gated sodium channel intracellular domain fused to yellow fluorescent protein (YFP-Na_V_II–III) fulfilled all of our criteria for successful live AIS label: this construct specifically localized to the AIS, accurately revealed plastic changes at the structure within hours, and, crucially, did not alter normal cell firing properties. We therefore recommend this probe for future studies of live AIS plasticity *in vitro* and *in vivo*.

## Introduction

In neurons, the axon initial segment (AIS) is a molecularly-defined portion of the proximal axon with unique structural and functional properties. It serves as a barrier that maintains distinct somatodendritic vs. axonal neuronal polarity (Rasband, 2010), and is a key regulator of neuronal excitability—in almost all neuronal cell types, and under almost all circumstances, the AIS is the site of action potential initiation (Bender and Trussell, 2012; Kole and Stuart, 2012).

It is also a highly dynamic structure. Over short-term timescales, the AIS can be modified by intrinsic conductances and intracellular signaling pathways, as well as by extrinsic synaptic and neuromodulatory inputs, to alter the initiation, patterning and spike waveform features of action potential firing (Bender et al., 2010, 2011; Grubb et al., 2011; Cotel et al., 2013; Martinello et al., 2015). Over longer timescales of hours to days, the structural and positional features of the AIS can also undergo modifications in response to sustained perturbations in neuronal activity. These structural forms of AIS plasticity—which can include changes in AIS length, position and/or ion channel distribution in both excitatory and inhibitory neurons (Grubb and Burrone, 2010a; Kuba et al., 2010; Muir and Kittler, 2014; Chand et al., 2015; Evans et al., 2015; Wefelmeyer et al., 2015)—have been shown to be associated with changes in neuronal excitability, and may form part of a repertoire of compensatory mechanisms acting to maintain network activity within set limits.

However, the evidence for structural AIS plasticity has thus far been limited to static snapshots, where plasticity is revealed *post hoc* by comparing fixed AIS label in separate groups of neurons subjected to different activity manipulations. Put simply, no individual AIS has ever been observed to change. This is despite the many potential benefits to be gained from live imaging of structural AIS plasticity. Following AISs live over time would allow us to definitively reveal local structural plasticity in individual neurons. It would also reduce the effects of cell-to-cell and experiment-to-experiment heterogeneity, permitting the detection of fine-scale changes that can be obscured in all but the largest of independent group datasets. It would allow studies of AIS plasticity to be combined with simultaneous live interrogation of neuronal function via electrophysiological and/or functional imaging techniques. And, finally, it has the potential to give us new insight into the mechanisms by which AIS plasticity is produced.

Here we characterize five alternative methodological approaches designed to live-label the AIS for timelapse imaging of activity-dependent plasticity. We find that, unlike other immunofluorescence-based and genetically-encoded probes, the fluorescently-tagged sodium channel motif YFP-Na_V_II–III meets our three criteria for a successful AIS live-label tool: (1) it accurately labels AIS structure under baseline conditions; (2) it reveals hours-scale AIS structural plasticity; and (3) it leaves neuronal excitability unperturbed.

## Materials and Methods

### Dissociated Cultures

Humane killing for tissue collection conformed to local King’s College London ethical approval under the UK Supplementary Code of Practice, The Humane Killing of Animals under Schedule 1 to the Animals (Scientific Procedures) Act 1986. Hippocampi were rapidly dissected from embryonic day (E18) Wistar rat embryos (Charles River) of either sex in ice-cold Hank’s balanced salt solution (HBSS). Tissue was trypsin digested (Worthington, 0.5 mg/ml; 15 min at 37°C), then triturated by repeatedly pipetting the cells using fire-polished Pasteur pipettes, and finally plated at a density of ~230 cells/mm^2^ on 13 mm coverslips (45,000 cells/coverslip; VWR) coated with poly-l-lysine (50 μg/ml, Sigma) and laminin (40 μg/ml). Cells were incubated at 37°C with 5% CO_2_ in Neurobasal medium containing 1% B27, 1% foetal calf serum and 500 μM Glutamax. At 4 days *in vitro* (DIV) half the media was changed with Neurobasal plus 2% B27 and 500 μM Glutamax. At 7 DIV media was topped up to 1 ml (13 mm coverslips) with fresh Neurobasal plus 2% B27 and 500 μM Glutamax. All experiments were carried out between 10–14 DIV. Unless otherwise stated, all cell culture reagents were obtained from Invitrogen.

### Depolarization Treatment

We carried out all treatments and analyses blind to experimental group. Depolarization was induced using the same protocol described by Evans et al. (2015). Briefly, neurons were treated in fully conditioned media by adding 15 mM KCl, or 15 mM NaCl as an osmolarity control.

### AIS Live-Labeling

#### Antibody

The mouse anti-pan-neurofascin antibody (extNF; A12/18, Neuromab) was generated against the rat-specific extracellular domain common to NF155 and NF186 (amino acids 25-1110). Our labeling protocol was very similar to that employed by Evans et al. (2015; see Table 1). Briefly, cells were pre-incubated for 5 min in 50:50 conditioned media: fresh Neurobasal, and 50 μM APV (NB-APV, Invitrogen) to protect against cell death (Hogins et al., 2011). After this, coverslips were briefly washed three times in NB-APV, after which they were placed in primary antibody solution (extNF, 1:200) diluted in NB-APV for 30 mins at 37°C. Next, cells were washed 3× in Neurobasal after which they were placed in secondary antibody (anti-mouse 488, 1:500, Invitrogen) diluted in NB-APV, for 1 min at room temperature (RT). After three final Neurobasal washes coverslips were transferred to the fully conditioned media in which they were situated prior to antibody labeling. For a subset of experiments we used an extNF antibody pre-bound with an Alexa488 antibody labeling kit (Thermo Fisher).

**Table 1:** **List of publications that have used extNF as a live axon initial segment (AIS) marker**.

Study	Cell culture source	Culture age	extNF ab conc.	Incubation time	2° ab conc.	Incubation time
Hedstrom et al. (2008)	E18 rat hippocampus	10 DIV	Not specified	30 min at 4°C	Not specified	Not specified
Schafer et al. (2009)	E18 rat hippocampus	10 DIV	1:200	30 min at 37°C	1:500	30 min at 37°C
Lukinavičius et al. (2014)^1^	P0–P1 rat hippocampus	Not specified	1:100	5 min at RT	Not specified	30 s at RT
Evans et al. (2015)	E18 rat hippocampus	10 DIV	1:200	3 min at 37°C	1:500	10 s at RT
Muir and Kittler (2014)^2^	E18 rat hippocampus	10 DIV	1:100	8 min at RT	1:350	8 min at RT
Current project	E18 rat hippocampus	10 DIV	1:200	30 min at 37°C	1:500	1 min at 37°C

*^1^D’Este et al. (2015) used the same live-labeling protocols. ^2^For this experiment the primary and secondary antibody mix were pre-incubated for 15 min on ice, after which they were applied to cells in an imaging media buffer containing 10% horse serum*.

#### Genetically-Encoded Fluorescent Probes

All genetically-encoded means of live-labeling the AIS were achieved via transfections with lipofectamine 2000 (Thermo Fisher), usually at 7 DIV (see Table 2). On the day of transfection, half of the conditioned cell media was removed from each well, after which cells were topped up with Neurobasal containing 2% B27 and 500 μM Glutamax, and placed back in the incubator (5% CO_2_, 37°C; 1 ml total volume) for a minimum of 30 min. The transfection solution mix was made by pre-incubating DNA constructs together with lipofectamine 2000 (0.5 μl/well) for 30 min at RT in Optimem media. Next, cells were treated with the DNA/lipofectamine 2000 mix (50 μl/well) for 10 or 5 min at 5% CO_2_, 37°C. Post transfection, cells were kept in a 1:1 solution containing conditioned cell media and Neurobasal containing 2% B27 and 500 μM Glutamax, usually until 10–12 DIV (see Table 2).

**Table 2:** **Transfection protocols for genetically-encoded AIS markers**.

DNA plasmid	Culture age: transfection—fixation	Transfection duration at 37°C	DNA concentration/coverslip	AIS localization?
YFP-Na_V_II–III	7–10 DIV	10 min	0.5 μg/ml	Yes

270kDa-AnkG-GFP	7–10 DIV	10 min	0.5 μg/ml	Yes

NF186-GFP	7–11 DIV	10 min	0.5 μg/ml	No
	7–14 DIV			No
	4–10 DIV			No
	4–14 DIV			No

Na_V_β4FL-GFP	7–10 DIV	10 min	0.5 μg/ml	No
			0.1 μg/ml	No
			0.3 μg/ml	No
		5 min	0.3 μg/ml	No
	11–14 DIV	10 min	0.3 μg/ml	No
			0.5 μg/ml	No
	11–12 DIV	10 min	0.1 μg/ml	No
			0.2 μg/ml	No

Na_V_β4−ΔCT-GFP	7–10 DIV	10 min	0.5 μg/ml	No
			0.3 μg/ml	No
			0.1 μg/ml	No
		5 min	3 μg/ml	No
	11–14 DIV	10 min	0.3 μg/ml	No
			0.5 μg/ml	No

The full-length rat neurofascin-186 construct expressing EGFP at the COOH terminus and a concomitant HA tag (NF186-GFP) was a gift from Matt Rasband (Baylor College of Medicine). The DNA plasmids containing the rat sodium channel β4 subunit fused with GFP at the C-terminal tail (full sequence, Na_V_β4-FL-GFP, C-terminal tail deletion Na_V_β4-ΔCT-GFP, gifts from the Rasband lab) were previously described by Buffington and Rasband (2013). The full-length rat 270kDa Ankyrin-G (AnkG) fused with EGFP at the COOH tail (270kDa-AnkG-GFP; a gift from Vann Bennett, Duke University) was originally described by Zhang and Bennett (1998). We excised the CMV promoter driving expression of the original 270kDa-AnkG-GFP plasmid via a restriction digest with AseI and EcoRI, and replaced it with the neuron-specific synapsin promoter.

The rat sodium channel 1.2 (Na_V_1.2) loop between the 2nd and the 3rd transmembrane domains fused to YFP at the C-terminus (YFP-Na_V_II–III) was subcloned via the following steps:

1. A synapsin promoter-driven YFP construct was made from the ChR2-YFP-Na_V_II–III plasmid previously described in Grubb and Burrone (2010b; Addgene deposit #26057) by using the 5′ primer CTACCGGTGCCACCATGGTGAGCAAGGGCGAGGAGCTGTTCA together with a 3′ primer TCGAATTCTTACTTGTACAGCTCGTCCATGCCG to PCR clone the YFP region. Next, the ChR2-YFP-Na_V_II–III region from another sample of ChR2-YFP-Na_V_II–III plasmid was excised, creating a synapsin promoter-only backbone construct which was then ligated with the YFP sequence.
2. The synapsin-YFP-Na_V_II–III construct used here was made by first excising the Na_V_II–III region via a Bsrg1 digest of a CMV promoter-driven YFP-Na_V_II–III plasmid (Addgene deposit #26056). Next, this region was blunt ligated with a Bsrg1-linearized synapsin promoter-driven YFP plasmid.

### Immunocytochemistry

Cells were fixed immediately after 3 h or 6 h treatment in 4% paraformaldehyde (PFA, TAAB Laboratories; in 3% sucrose, 60 mM PIPES, 25 mM HEPES, 5 mM EGTA, 1 mM MgCl_2_) for 20 min at RT. Permeabilization was carried out in 0.25% Triton X-100 (Sigma) in PBS for 5 min at RT and the block step was carried out in 10% Normal Goat Serum (NGS, Sigma) in PBS for 1 h at RT. 1° antibody incubation was in 2% NGS in PBS for 1 h at RT with the following antibodies: rabbit anti-prox1 (1:1000, Sigma), mouse IgG1 or mouse IgG2B anti-AnkG (1:500, N106/43 and N106/65, Neuromab). This was followed by 5× PBS washes, after which coverslips were incubated with the appropriate Alexa Fluor 2° antibody for 1 h at RT: anti-rabbit 594, anti-mouse IgG1 or IgG2B 633. Stained coverslips were mounted on glass slides with Mowiol (Calbiochem).

### AIS Imaging and Analysis

The transfection efficiency of our genetically encoded AIS live-label constructs was not uniform. For this reason, we selected only DGCs which displayed a high, AIS-specific increase of fluorescence signal with a low overall signal in the soma, dendrites and the rest of the axon. We did not analyze cells in which the construct was over-expressed indiscriminately throughout the entire extent of the neuron. Once a suitable cell was identified, all images were obtained with a Zeiss LSM 710 confocal microscope, using a 40× oil immersion objective and appropriate laser excitation and filters. Image size was 512 × 512 pixels, with 0.138μm/pixel XY resolution, and Z steps of 0.664 μm. *Z*-stack images were then converted into maximum intensity projections and exported into Matlab (Mathworks) for AIS length measurements using custom-made scripts (Evans et al., 2015; freely available at Matlab Central). AIS length was calculated by measuring fluorescence intensity along a line drawn by hand starting at the soma, down the axon, through and past the AIS. Fluorescence intensity measures were averaged over a 3 × 3-pixel square centered on the pixel of interest. Averaged profiles were smoothed using a ~5 μm sliding mean and normalized between 1 and 0. AIS start and end positions were the proximal and distal axonal locations where the normalized, smoothed profile declined to 0.33. AIS length was calculated as the axonal distance between the start and end positions. For all experiments where an AIS was labeled with two different markers, e.g., YFP-Na_V_II–III and AnkG, AIS lengths for both labels were obtained concomitantly from the same axonal drawn line profile.

### Electrophysiology

Whole-cell patch-clamp recordings were obtained from both untransfected and YFP-Na_V_II–III + Tag-RFP-expressing DGCs identified based on morphology (Evans et al., 2013, 2015) at 10–12 DIV. Cells were patched at RT in an HBS extracellular solution (pH 7.4, ~290 mOsm) which contained: 136 mM NaCl, 2.5 mM KCl, 10 mM HEPES, 10 mM _D_-glucose, 2 mM CaCl_2_, 1.3 mM MgCl_2_, 0.01 mM SR-95531 (gabazine, Sigma), 0.02 mM NBQX and 0.025 mM APV. Pipettes with a 3–7 MΩ resistance were pulled from borosilicate glass (1.17 mm inner diameter, 1.5 mm outer diameter, Harvard Apparatus), fire polished (Narishige microforge) and filled with an internal solution that contained: 130 mM K-gluconate, 10 mM NaCl, 1 mM EGTA, 0.133 mM CaCl_2_, 2 mM MgCl_2_, 10 mM HEPES, 3.5 mM NaATP, 1 mM NaGTP (pH 7.4, ~290 mOsm). Signals were measured with a Heka EPC10 amplifier coupled to Patchmaster software. Signals were Bessel filtered at 10 kHz (filter 1) and 2.9 kHz (filter 2, active filters used in voltage-clamp only), digitized and sampled at 20–200 kHz (5–50 μs sample interval) depending on the protocol. Fast capacitance was compensated in the on-cell configuration, slow capacitance was compensated after membrane rupture, and 100% bridge balance was employed during current-clamp recordings. Data are uncorrected for an estimated liquid junction potential of ~15 mV.

Series resistance (*R*_s_) was calculated as a cell’s response to a 10-mV hyperpolarization step in voltage clamp with slow capacitance compensation disabled. Membrane resistance (*R*_m_) was obtained from the steady holding current at the new step, and membrane capacitance (*C*_m_) was the area under the exponentially decaying current from peak to holding. Series resistance was used as a proxy for patch quality as only cells with *R*_s_ < 25 MΩ were selected for analysis. *R*_s_ was measured several times during the recording session and any data in which the following *R*_s_ varied by more than 20% from the initial value were excluded. To look at single spike properties we evoked spikes in current-clamp mode at *V*_hold_ −60 ± 3 mV. For action potential waveform measures, we injected 10-ms-duration current steps of increasing amplitude until we reached the current threshold at which the neuron reliably fired an action potential (*V*_m_ > 0 mV). For multiple spiking measures, we injected 500-ms current steps of increasing amplitude with 2-s inter-sweep interval until the neuron passed its maximum spike number.

Exported traces were analyzed using custom-written MATLAB routines. To determine voltage threshold, 5-μs sample interval recordings of spikes fired at threshold 10-ms current injection were smoothed using a 20-point (100-μs) sliding filter, before differentiation for dV/dt. Voltage threshold was taken as the unsmoothed potential at which dV/dt first passed 10 V/s. AP height was calculated as the difference between *V*_max_ and *V*_threshold_. Spike width was measured at the midpoint between *V*_threshold_ and V_max_.

### Statistics

Statistical analysis was carried out with Prism (Graphpad) and SPSS (IBM). Sample distributions were first assessed for normality with the D’Agostino and Pearson omnibus test. Details regarding the specific parametric or non-parametric tests carried out are reported in the results section. α values were set to 0.05 unless otherwise stated, and tests were two-tailed for all experiments.

## Results

**Figure 1:**
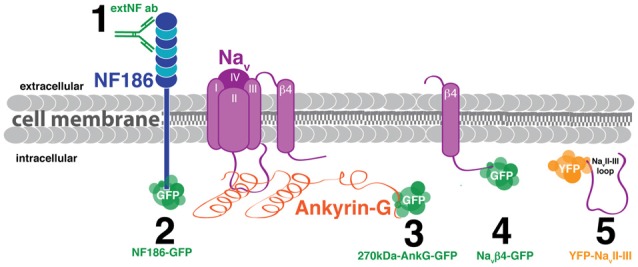
**Schematic of candidate methods for live labeling the axon initial segment (AIS) *in vitro*. (1)** Antibody targeted against the neurofascin extracellular domain (extNF ab). **(2)** Plasmid DNA fusion construct of neurofascin and GFP (NF186-GFP). **(3)** Plasmid DNA fusion construct of 270kDa-ankyrin-G (AnkG) and GFP (270kDa-AnkG-GFP). **(4)** Plasmid DNA fusion construct of the sodium channel β4 subunit and GFP (Na_V_β4-GFP). **(5)** Plasmid DNA fusion construct of the sodium channel loop between the II and III α subunits and YFP (YFP-Na_V_II–III).

We screened several candidate approaches for live imaging of structural plasticity at the AIS. Key criteria for successful live label with a given probe were that it revealed AIS structure accurately—even after activity-dependent plasticity—and that it did not itself alter AIS structure or function. In other words, we were looking for a probe that was accurate, plastic, and benign. We trialed five separate probes in dissociated cultures of rat hippocampal cells: one based on immunocytochemistry, and four that were genetically-encoded (Figure 1). To reduce cell-type heterogeneity, we focused exclusively on DGCs. These hippocampal glutamatergic projection neurons retain many of their distinctive morphological and functional features in dissociated culture, and can be readily identified either on the basis of gross morphology or by post-fixation immunocytochemical label for the transcription factor prox1 (Williams et al., 2011; Evans et al., 2013, 2015; Lee et al., 2013).

### Live AIS Label with a Neurofascin Antibody Is Accurate at Baseline but Does Not Report Rapid Structural Plasticity

An immunohistochemical approach to labeling the AIS in live neurons has clear theoretical benefits. It has the potential to provide comprehensive label of every AIS in a given sample, and, unlike genetically-encoded probes, can be achieved in minutes without invasively introducing any foreign intracellular material (Schafer et al., 2009; Evans et al., 2015). It does, though, rely on the availability of an antibody that recognizes an extracellular epitope of an AIS-localized protein. Here we used a rat-specific monoclonal antibody designed to recognize the extracellular domain of neurofascin isoforms 186 (found at the AIS and the node of Ranvier) and 155 (found at the paranode; Tait et al., 2000). This “extNF” antibody has been previously employed to reveal baseline AIS position and length in live dissociated rat neurons (Hedstrom et al., 2008; Lukinavičius et al., 2014; Muir and Kittler, 2014; D’Este et al., 2015; Evans et al., 2015), and also to report live injury-induced alterations in AIS structure (Schafer et al., 2009; Table 1).

**Figure 2:**
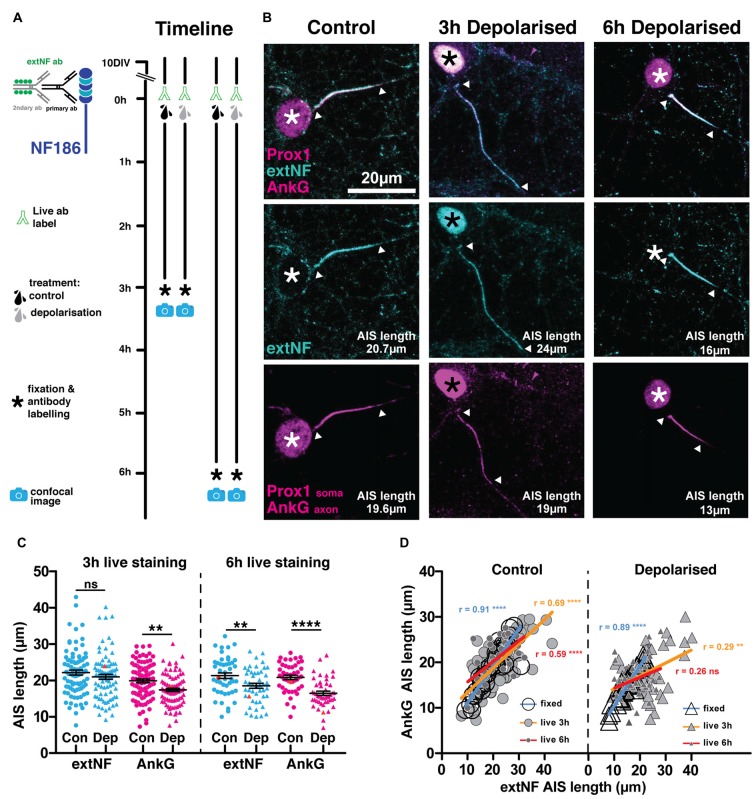
**Live AIS label with a neurofascin antibody does not report rapid structural plasticity. (A)** Diagram of the experimental timeline used to test the extNF antibody as an AIS live label method. **(B)** Example maximum intensity projections of control or 3 h/6 h depolarized dentate granule cells (DGCs) bearing extNF live-labeled AISs (cyan) and stained post fixation with antibodies against AnkG (magenta, axonal) or prox1 (magenta, nuclear, white asterisks; cyan nuclear, black asterisks—due to secondary antibody contamination); arrowheads, DGC AIS start and end positions. **(C)** Plot shows distributions of AIS lengths measured with extNF applied live for either 3 or 6 h (blue) and AnkG (magenta) from control (Con) or depolarized (Dep) DGCs. Each point represents a single cell; black lines show mean ± SEM; orange symbols, values from example cells displayed in **(B)**; Bonferroni post-test after two-way repeated measures ANOVA; ns, non-significant; **p < 0.01; ****p < 0.0001. **(D)** Correlation analysis for AISs labeled with both extNF and AnkG in the same neuron under control (dotted symbols) or depolarized (triangle symbols) conditions in three separate experiments: extNF post fixation (large open circles and triangles), extNF applied live for the duration of a 3 h treatment (medium sized light gray filled dots and triangles), extNF applied live for the duration of a 6 h treatment (small, dark gray filled dots and triangles). Lines show best linear regression fit for extNF applied post fixation (blue), live for 3 h (orange) or live for 6 h (red); Pearson’s correlation; **p = 0.008; ****p < 0.0001; ns, non-significant.

**Figure 3:**
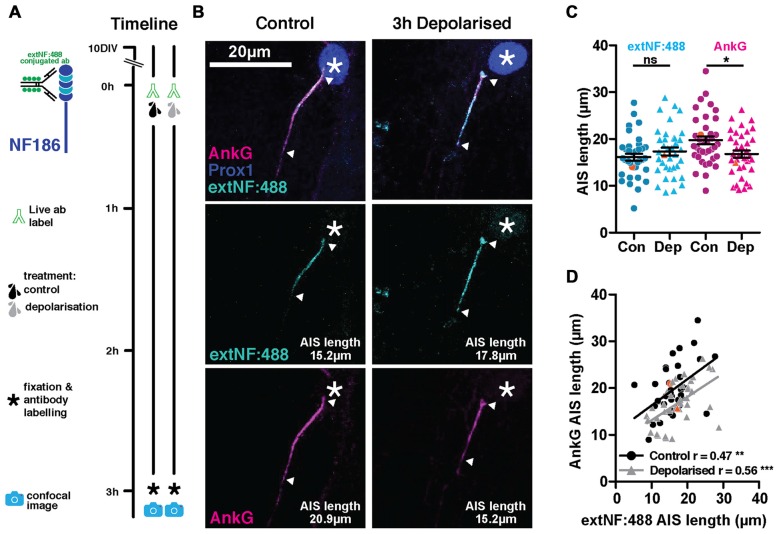
**ExtNF antibody conjugated with Alexa-488 does not track AIS plasticity. (A)** Diagram of AIS live label method used and experimental timeline. **(B)** Maximum intensity projections of example neurons labeled live with the extNF antibody conjugated to Alexa-488, after which they were subjected to either a 3 h control or depolarization treatment. Following fixation, cells were stained with antibodies against AnkG (magenta) and Prox1 (dark blue). Asterisks, nucleus; arrowheads, DGC AIS start and end positions. **(C)** Distributions of AIS lengths measured with extNF-488 conjugated antibody and AnkG from control or depolarized DGCs. Each dot or triangle represents a single cell; orange symbols, values from example cells displayed in **(B)**; dark lines show mean ± SEM; Bonferroni post-test after two-way repeated measures ANOVA; ns, non-significant; *p < 0.05. **(D)** Correlation analysis for AISs labeled with both the extNF-488 conjugated antibody label and AnkG under control or depolarized conditions. Orange symbols, values from example cells displayed in **(B)**; lines show best linear regression fit for control (black) or depolarized cells (gray); **p < 0.01; ***p < 0.001.

However, although extNF label is a reasonably accurate indicator of baseline AIS features in live neurons, we found that it was unable to follow rapid structural AIS plasticity. As an initial test of this probe’s suitability, we live-labeled 10 DIV hippocampal cells with extNF and an appropriate Alexa-conjugated secondary antibody, then induced chronic depolarization for 3 h with elevated external potassium (+15 mM KCl, Figure 2A). In previous comparisons vs. control cells treated with +15 mM NaCl, this manipulation produced rapid structural AIS plasticity: post-fix immunocytochemical labeling of the key AIS scaffolding molecule AnkG, of voltage-gated sodium channels (Pan-Na_V_), or of neurofascin using extNF itself, all consistently revealed a ~5 μm (25%) reduction in DGC AIS length (Evans et al., 2015). Indeed, in DGCs live-labeled with extNF throughout the treatment period we again observed this rapid form of structural AIS plasticity, with AnkG distributions significantly shorter in depolarized compared to control neurons (Figures 2B,C; 3 h treatment two-way repeated-measures ANOVA; treatment, *F*_(1,350)_ = 9.02 *p* = 0.0029; label type, *F*_(1,350)_ = 23.57 *p* < 0.0001; interaction, *F*_(1,350)_ = 1.41 *p* = 0.24; Bonferroni post-test AnkG control vs. depolarized *t* = 2.96 *p* < 0.01). However, in the very same cells the shortening effect was not observed in the live-applied extNF label distribution (Figures 2B,C left panel, Bonferroni post-test extNF control vs. depolarized *t* = 1.28 *p* > 0.05).

Assessing correlations between AnkG and live-applied extNF label on a cell-by-cell basis further underscored this mismatch under plastic conditions. When both antibodies were applied post-fixation, AIS lengths measured from AnkG and extNF label correlated extremely well in both control and depolarized DGCs (Evans et al., 2015; Figure 2D; controls Spearman’s *r* = 0.91, *p* < 0.0001, *n* = 37; depolarized Spearman’s *r* = 0.89, *p* < 0.0001, *n* = 35). A significant correlation was still present for control cells when the extNF label was applied throughout the 3 h +15 mM NaCl treatment, but was weaker than for dual fixed label (Figure 2D; extNF vs. AnkG in control cells Spearman’s *r* = 0.69, *p* < 0.0001, *n* = 92). This shows that extNF label provides a less accurate picture of AIS structure when applied to live neurons, an unsurprising result given the shorter antibody incubation times and lack of blocking step that are necessary in this protocol. In 3 h depolarized cells, however, the correlation between AnkG- and live-extNF-defined AIS lengths was even weaker (Figure 2D right panel; extNF vs. AnkG in depolarized cells Spearman’s *r* = 0.29, *p* < 0.0080, *n* = 85), providing further evidence that this live labeling approach cannot be used to accurately report structural AIS plasticity.

We reasoned that live extNF immunolabel might still be capable of revealing AIS plasticity over longer timescales. We therefore live-labeled 10 DIV cultures with extNF before exposing them to extended 6 h treatments with +15 mM NaCl or KCl. Similar to 3 h manipulations, 6 h depolarization was again associated with significantly shorter DGC AISs measured with post-fixation AnkG label (Figures 2B,C right panel; 6 h treatment two-way repeated-measures ANOVA; treatment, *F*_(1,91)_ = 19.98 *p* < 0.0001; label type, *F*_(1,91)_ = 6.43 *p* < 0.013; interaction, *F*_(1,91)_ = 2.502 *p* = 0.12; Bonferroni post-test AnkG control vs. depolarized *t* = 4.63 *p* < 0.0001). Now, 6 h-depolarized DGCs also had significantly shorter AISs than their control counterparts as assessed by live extNF label (Figure 2C right panel; Bonferroni post-test extNF control vs. depolarized *t* = 2.95 *p* < 0.01). However, the live extNF axonal distributions did not shorten as much as the AnkG distributions in the same neurons, to the extent that the correlation between the two labeling approaches was no longer significant in the depolarized group (Figure 2D right panel; depolarized cells extNF vs. AnkG Spearman’s *r* = 0.25, *p* = 0.0798, *n* = 47). Even after more prolonged perturbations of neuronal activity, then, live-applied extNF label cannot provide an accurate representation of plastic changes in AIS structure.

Live immunolabeling approaches using standard, multivalent probes suffer the potential risk of crosslinking antigen molecules at the levels of both primary and secondary antibodies, thereby altering mobility or function of the bound antigen. If neurofascin molecules live-labeled with extNF were somehow rendered more stable by such crosslinking, this may explain the inaccuracy of the approach in labeling plastic changes in AIS structure. We therefore ruled out the possibility of cross-linking at the secondary antibody level by pre-conjugating the extNF primary antibody with an Alexa 488 fluorophore before applying it to our neurons in a single labeling step (Figure 3A). However, this pre-conjugated extNF-488 live label was no more accurate in reporting rapid activity-dependent AIS shortening. While AnkG-defined DGC AIS lengths were again significantly shorter in the 3 h-depolarized group, distributions of pre-conjugated, live-applied extNF-488 label did not differ between treatment groups (Figures 3B,C; two-way repeated measured ANOVA; treatment, *F*_(1,73)_ = 0.77 *p* = 0.38; label type, *F*_(1,73)_ = 7.42 *p* = 0.008; interaction *F*_(1,73)_ = 13.73 *p* = 0.0004; Bonferroni post-test, extNF-488 control vs. depolarized treatments *t* = 1.07 *p* > 0.05; AnkG control vs. depolarized treatment *t* = 2.6 *p* < 0.05). Moreover, correlations between AIS length measured by AnkG and pre-conjugated extNF-488 label were relatively weak in both control and depolarized cells (Figure 3D; control cells Pearson’s *r* = 0.47 *p* = 0.003 *n* = 38; depolarized cells Pearson’s *r* = 0.56 *p* = 0.0003 *n* = 37).

So, despite the potential advantages of live-labeling the AIS with an immunocytochemical approach, and despite the fact that extNF label might reveal some aspects of AIS plasticity under longer activity perturbations, for an accurate readout of structural AIS changes in live neurons we needed to consider alternative, genetically-encoded strategies.

### Lack of AIS Label with Neurofascin-186-GFP or Na_V_β4-GFP

We tested four genetically-encoded probes for their potential to live-label the AIS. For two of these constructs—fusion proteins of GFP with full-length neurofascin-186 or the Na_V_β4 subunit—we were unfortunately unable to find the experimental conditions for successful AIS localization.

**Figure 4:**
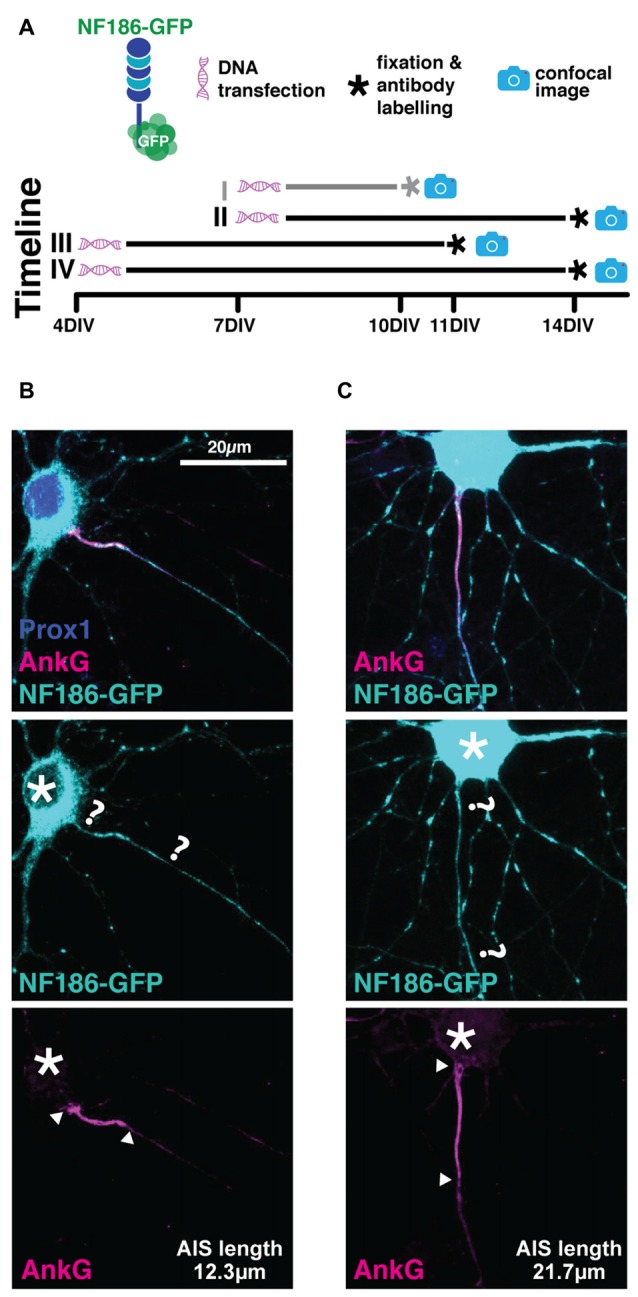
**NF186-GFP does not localize specifically to the AIS. (A)** Diagram of experimental timeline for all the four transfection methods attempted (see Table 2); gray line highlights method used for the cells displayed in panel **(B,C)**. **(B)** Maximum intensity projection of the best observed co-localization between the NF186-GFP construct (cyan) and AnkG (magenta). Asterisks, soma; question marks, putative start and end of NF186-GFP-labeled AIS; arrowheads, AnkG-labeled AIS start and end positions. **(C)** As in **(B)**, but showing the most commonly observed expression pattern of NF186-GFP.

We used a rat-specific, full-length neurofascin-186 construct tagged with GFP at its C-terminal domain (NF186-GFP), first described by Zhang and Bennett (1998) and used more recently by Dzhashiashvili et al. (2007). We reasoned that over-expression of NF186-GFP starting at a stage at which most AISs have already been established in culture (7 DIV) should not disrupt AIS assembly or disturb cell function. To test its expression pattern we initially transfected cells according to our standard lipofection protocol at 7 DIV (see “Materials and Methods” Section; Table 2), after which at 10 DIV we fixed and stained with an antibody against AnkG to check co-localization with endogenous AIS labeling, and prox1 to confirm DGC identity. We also ran several trials in which: (1) the transfection protocol was started earlier at 4 DIV, and (2) allowed cells a longer developmental time until 14 DIV (Figure 4A; Table 2). In all experiments, we failed to see precise co-localization between NF186-GFP and AnkG antibody label (Figure 4). The NF186-GFP signal tended to be strongly expressed in the cell soma and in a punctate fashion across the dendrites and the axon (Figure 4). Even in the best example of AnkG co-localization, NF186-GFP did not specifically localize to the AIS (Figure 4B), thereby failing to fulfil our first condition as a suitable AIS live-label.

**Figure 5:**
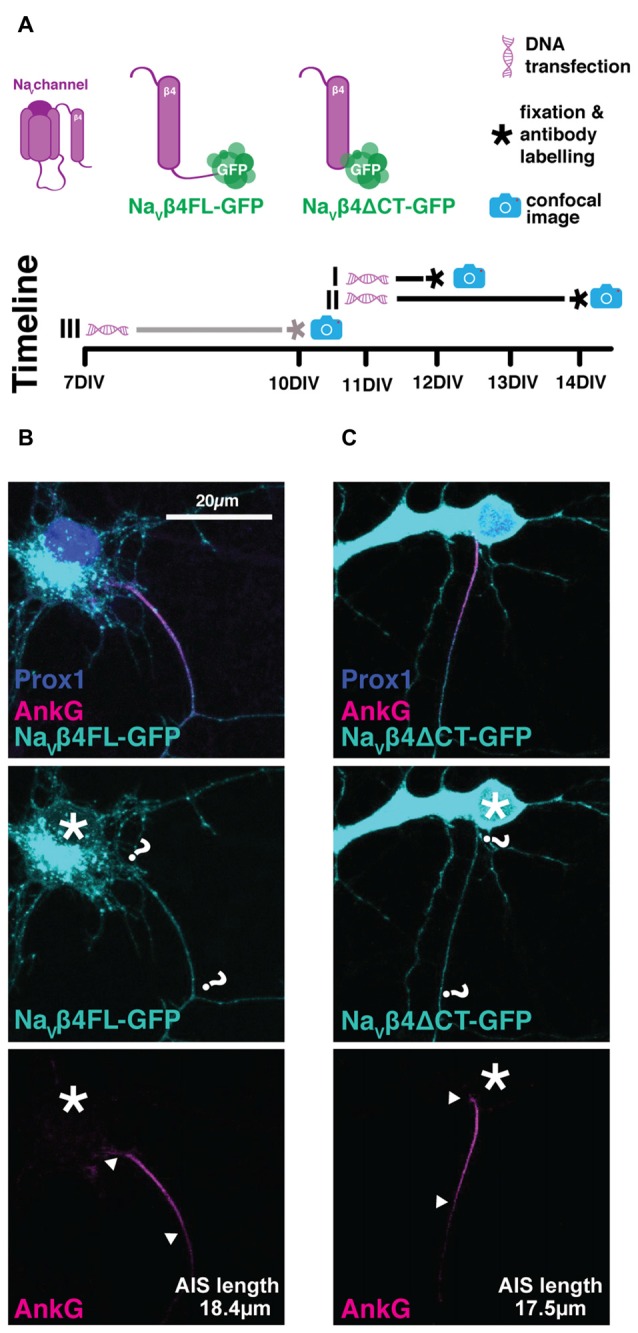
**Lack of AIS specificity with the Na_V_β4-GFP constructs. (A)** Diagram of experimental timeline for the three transfection methods attempted (see Table 2); gray line highlights method used for the cells displayed in panels **(B,C)**. **(B)** Maximum intensity projection of the usual expression pattern of Na_V_β4FL-GFP (cyan) and lack of co-localization with AIS marker AnkG (magenta). Asterisks, soma; question marks, presumptive location of Na_V_β4-GFP AIS; arrowheads, AnkG AIS start and end positions. **(C)** As in **(B)**, but for the Na4ΔCT-GFP construct.

Na_V_β4 is an auxiliary sodium channel subunit, thought to be responsible for the resurgent Na^+^ current through the action of its C-terminal tail as an open-channel blocker (Grieco et al., 2005; Bant and Raman, 2010). Due to the high concentration of sodium channels found at the AIS, it was previously shown that overexpression of Na_V_β4-GFP labels AISs in fixed tissue from multiple brain regions, including the hippocampus in both slices and dissociated *in vitro* cultures (Buffington and Rasband, 2013). We tested two constructs where Na_V_β4 was fused with GFP: (1) the full-length channel subunit (Na_V_β4-FL-GFP, Figure 5B) and (2) a version with a truncation of the functionally important C-terminal tail (Na_V_β4-ΔCT-GFP, Figure 5C). DGCs do not express Na_V_β4 (Yu et al., 2003; Castelli et al., 2007), so we reasoned that these constructs—especially the functionally null Na_V_β4-ΔCT-GFP version—might be a benign way of labeling AISs live. We therefore transfected cells with Na_V_β4-FL-GFP or Na_V_β4-ΔCT-GFP at several stages: either at 7 DIV followed by fixation at 10 DIV as per our normal protocol, or at 11 DIV followed by fixation at 12 or 14 DIV in order to match previously published expression methods (Buffington and Rasband, 2013, Figure 5; Table 2). All cells were immuno-labeled against AnkG and prox1 post fixation. Unfortunately, we were not able to see clear co-localization of either Na_V_β4-GFP probe with endogenous AnkG label. Regardless of the transfection protocol used, both constructs were strongly expressed in the cell soma and in a punctate fashion throughout dendrites and axons (Figure 5). We therefore concluded that—at least in our hands—neither of the Na_V_β4-GFP plasmids was suitable for use as a live AIS marker.

### Overexpression of 270kDa-AnkG-GFP Alters AIS Structure in Mature Neurons

**Figure 6:**
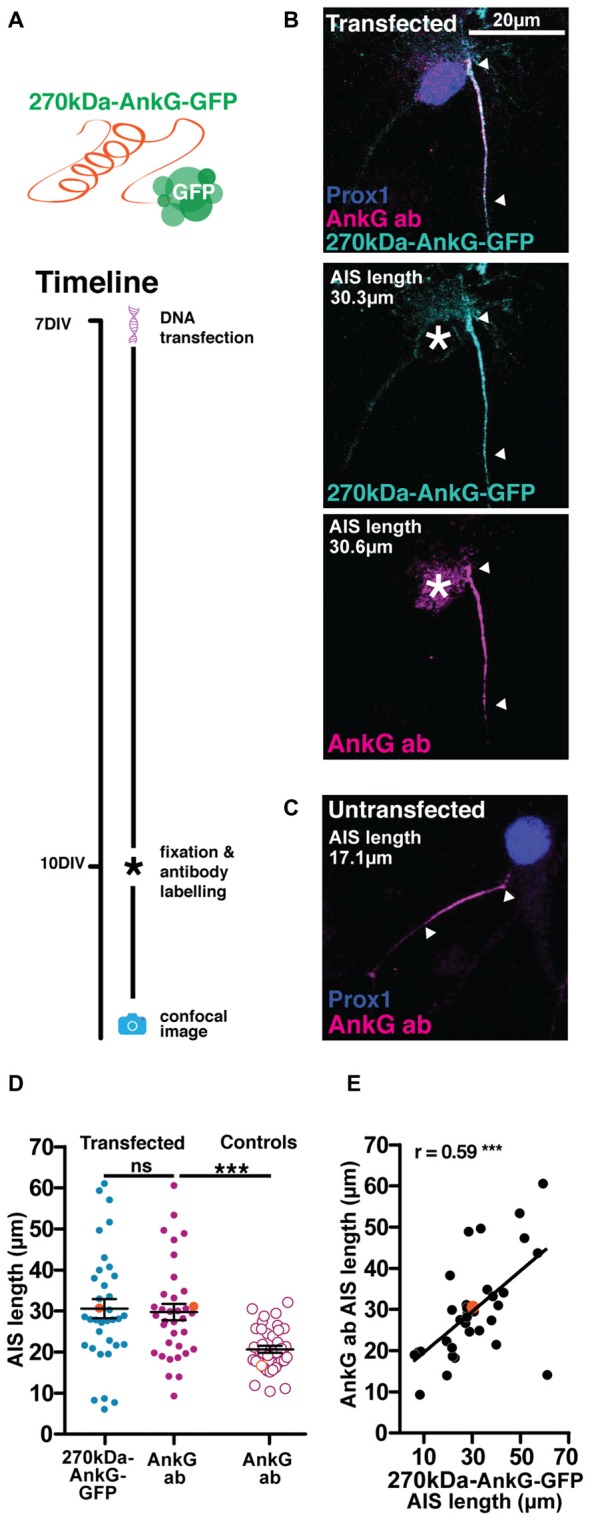
**The 270kDa-AnkG-GFP construct artificially increases AIS length in mature neurons. (A)** Diagram of construct used and transfection method timeline. **(B)** Maximum intensity projection of a DGC expressing 270kDa-AnkG-GFP (cyan) which co-localizes with an anti-AnkG antibody (magenta). Asterisks, nucleus; arrowheads, DGC AIS start and end positions. **(C)** Maximum intensity projection of an untransfected DGC bearing an AIS of normal length. Arrowheads DGC AIS start and end positions. **(D)** Distributions of AIS lengths measured from 270kDa-AnkG-GFP transfected neurons and control untransfected DGCs. Each dot or triangle represents a single cell; orange symbols represent cells from panel **(B)**; dark lines show mean ± SEM; paired *t*-test AnkG-GFP and AnkG ab, *p* = 0.68; ns, non-significant; unpaired *t*-test AIS length via AnkG ab from transfected vs. control untransfected cells, ***p = 0.002. **(E)** Correlation analysis for AISs labeled with both the AnkG-GFP construct and AnkG antibody. Each dot represents one cell; orange symbol, example cell from panel **(B)**; line; best fit linear regression; Pearson’s correlation; ***p < 0.001.

Our third genetically-encoded probe consisted of GFP fused to the C-terminus of the 270kDa isoform of AnkG (270kDa-AnkG-GFP). We were aware that overexpressing this AnkG isoform in very young neurons (Galiano et al., 2012) or in AnkG-null cells (Jenkins et al., 2015) was previously shown to produce abnormally elongated AISs. However, we reasoned that overexpressing 270kDa-AnkG-GFP in more mature wild-type neurons (Figure 6A; Table 2), in the presence of higher levels of endogenous AnkG and after the establishment of the AnkG-ankyrin-B boundary at the distal AIS (Galiano et al., 2012), might allow accurate live label of normal-length AISs. We also drove 270kDa-AnkG-GFP from the synapsin promoter, in order to achieve both neuronal specificity and activity-independent expression (see “Materials and Methods” Section). With this probe, localization to the AIS was good, with a significant correlation between AIS length revealed by the live marker and immunocytochemical label for total AnkG distribution (Figure 6E; 270kDa-AnkG-GFP vs. AnkG antibody Pearson’s *r* = 0.59 *p* = 0.0002 *n* = 35). However, under baseline conditions overexpression of 270kDa-AnkG-GFP from 7 DIV still produced a marked increase in AIS length when compared to neighboring non-transfected DGCs (Figures 6B–D; unpaired *t*-test on AIS AnkG antibody length from transfected vs. untransfected cells *t* = 4.1 *p* = 0.0001). Since this approach therefore broke our key requirement that live label should leave baseline AIS structure unperturbed, it was rapidly abandoned.

### YFP-Na_V_II–III Provides Accurate Live AIS Label That Is Both Plastic and Functionally Benign

**Figure 7:**
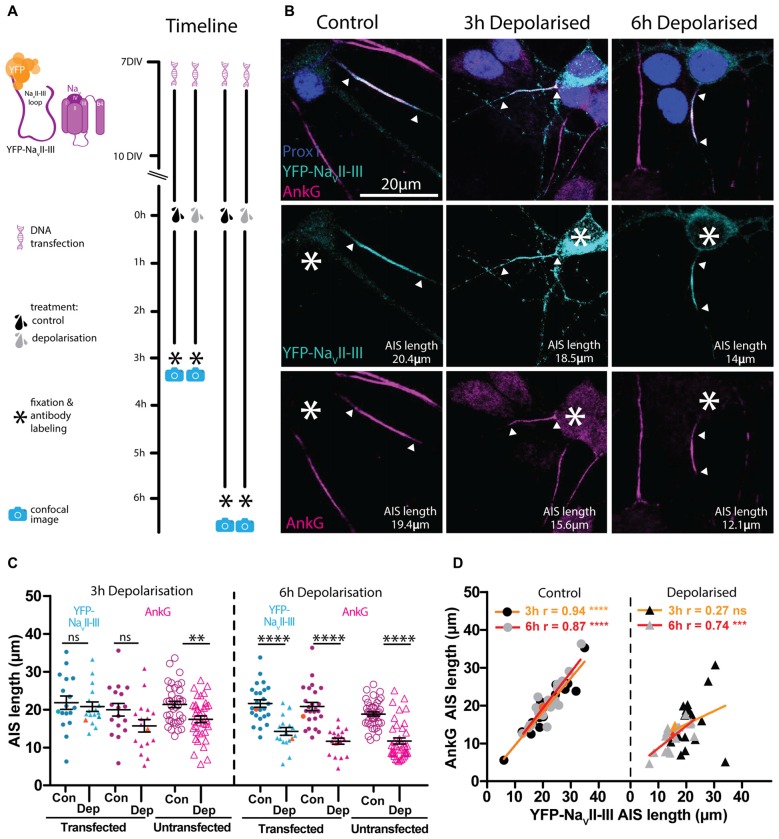
**YFP-Na_V_II–III has a high degree of correlation with AnkG and is able to accurately track AIS plasticity following a 6 h depolarization treatment. (A)** Diagram of the experimental timeline used to test the YFP-Na_V_II–III construct as an AIS live label. **(B)** Maximum intensity projections of control or 3 h and 6 h depolarized DGCs bearing YFP-Na_V_II–III live-labeled AISs (cyan) and stained post fixation with antibodies against AnkG (magenta) or prox1 (dark blue). Asterisks, soma; arrowheads, DGC AIS start and end positions. **(C)** Distributions of AIS lengths determined from YFP-Na_V_II–III (blue) and AnkG (magenta) from control (Con) or depolarized (Dep) DGCs. Each symbol represents a single cell; black lines show mean ± SEM; orange symbols represent cells from panel **(B)**; Bonferroni post-test after two-way repeated measures ANOVA; ns, non-significant; **p < 0.01; ****p < 0.0001. **(D)** Correlation analysis for AISs labeled with YFP-Na_V_II–III and AnkG under control (dotted symbols) or depolarized (triangle symbols) conditions. Lines show best linear regression fit for a 3 h (orange) or 6 h treatment (red); orange symbols represent cells from panel **(B)**; Pearson’s correlation; ns, non-significant; ***p < 0.001; ****p < 0.0001.

Rather than overexpressing fluorescently-tagged full-length AIS proteins, our final genetically-encoded strategy for live AIS label involved a highly conserved sub-region of vertebrate Na_V_ channels. The intracellular loop between Na_V_ transmembrane domains II and III (Na_V_II–III) is both necessary and sufficient for these channels to bind AnkG and localize to the AIS (Garrido et al., 2003; Lemaillet et al., 2003; Gasser et al., 2012), and under the control of the CMV promoter it can be used to accurately report long-term (48 h) activity-dependent changes in AIS position (Grubb and Burrone, 2010a). Here we employed this fusion protein under the control of the neuron-specific and non-activity-dependent synapsin promoter. Sparse expression in hippocampal neurons under control conditions resulted in YFP-Na_V_II–III specifically accumulating at the AIS, with markedly lower expression levels in the soma (Figures 7A,B). Live-labeled in this way, DGC AISs at baseline were of normal length and correlated extremely well with post-fixation immunocytochemical label for AnkG (Figure 7D left panel; 3 h control treatment YFP-Na_V_II–III post fix vs. AnkG antibody Pearson’s *r* = 0.94 *p* < 0.0001 *n* = 18). This probe therefore offers highly accurate labeling without morphological distortion of the AIS under baseline conditions.

We next asked if YFP-Na_V_II–III is also an accurate reporter of structural AIS plasticity. However, although 3 h depolarization with +15 mM KCl produced a significant reduction in AIS length revealed by post-fixation AnkG label in non-transfected cells, the YFP-Na_V_II–III distribution in neighboring transfected neurons showed no evidence of shortening (Figure 7C left panel; two-way repeated measured ANOVA; treatment, *F*_(1,33)_ = 1.99 *p* = 0.17; label type, *F*_(1,33)_ = 14.45 *p* = 0.0006; interaction *F*_(1,33)_ = 3.13 *p* = 0.09; Bonferroni post-test, YFP-Na_V_II–III transfected control vs. depolarized treatments *t* = 0.48 *p* > 0.05; AnkG AIS length in transfected control vs. depolarized cells *t* = 1.82 *p* > 0.05; AnkG AIS length in untransfected cells unpaired *t-test* control vs. depolarized *t* = 3.20 *p* = 0.0020 *n* = 72). With an extended 6 h depolarization treatment, though, the probe did accurately reflect structural AIS plasticity: the YFP-Na_V_II–III distribution was significantly shorter in 6 h-depolarized vs. control neurons (Figure 7C right panel; two-way repeated measures ANOVA; treatment, *F*_(1,41)_ = 35.47 *p* < 0.0001; label type, *F*_(1,41)_ = 15.37 *p* = 0.0003; interaction *F*_(1,41)_ = 4.2 *p* = 0.047; Bonferroni post-test, YFP-Na_V_II–III transfected control vs. depolarized treatments *t* = 5.08 *p* < 0.0001; AnkG transfected control vs. depolarized cells *t* = 6.29 *p* < 0.0001; AnkG AIS length in untransfected cells unpaired *t*-test control vs. depolarized *t* = 7.31 *p* < 0.0001 *n* = 76), and was strongly and significantly correlated with AnkG immunocytochemical label in both treatment groups (Figure 7D red lines; 6 h control treatment YFP-Na_V_II–III vs. AnkG antibody Pearson’s *r* = 0.87 *p* < 0.0001 *n* = 25; 6 h depolarized treatment YFP- Na_V_II–III vs. AnkG antibody Pearson’s *r* = 0.74 *p* = 0.0005 *n* = 18).

**Figure 8:**
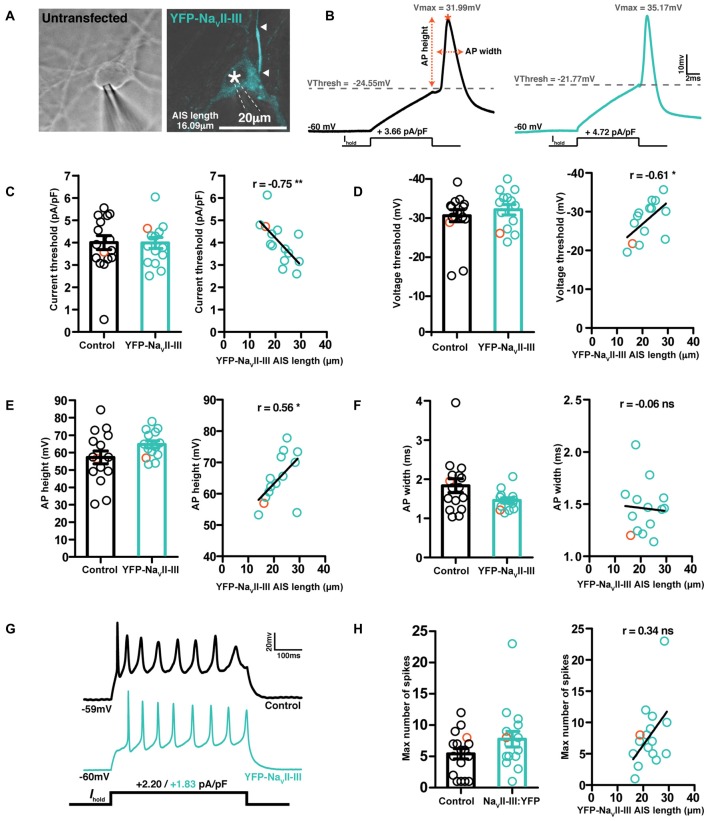
**YFP-Na_V_II–III labeled cells exhibit normal spike firing properties. (A)** Brightfield image of a control untransfected neuron, and a maximum intensity projection of a cell expressing YFP-Na_V_II–III (cyan), both patched in whole-cell mode. Asterisks, nucleus; arrowheads, DGC AIS start and end positions. **(B)** Example whole-cell current-clamp recordings of threshold APs fired to 10-ms somatic current injection from neurons displayed in **(A)**; orange asterisk, V max; orange dotted lines, AP height and width calculation. **(C)** Single action potential current threshold; left, each dot represents a single cell from either the control untransfected group (black) or YFP-Na_V_II–III expressing (cyan) group; orange colored symbols represent values from the example cells presented in panel **(A)**; bars show mean ± SEM; unpaired *t*-test *p* = 0.76. Right, correlation of current threshold vs. AIS length revealed by live label with YFP-Na_V_II-III; each dot shows one cell; orange dot shows the example cell from panel **(A)**; line shows best fit linear regression; **p < 0.01. **(D)** Single action potential voltage threshold; details same as panel **(C)**; left, *p* = 0.37; right, *p < 0.05. **(E)** Action potential height; details same as panel **(C)**; left, *p* = 0.1; right, *p < 0.05. **(F)** Action potential width at half height; details same as panel **(C)**; left, *p* = 0.1; right, ns, non-significant. **(G)** Example traces of maximum firing elicited via a 500 ms duration current injection used to probe repetitive spiking. **(H)** Maximum number of spikes fired to a 500 ms current injection pulse; details same as panel **(C)**; left, *p* = 0.2; right, ns, non-significant.

Over a slightly longer, but still hours-scale timeframe, YFP-Na_V_II–III is therefore an accurate reporter of activity-dependent structural change at the AIS. But, like all genetic probes, its use has the drawbacks of requiring the introduction of foreign material into neurons, a lack of temporal specificity, overexpression of biologically active proteins, and sparse expression. While the latter might be beneficial under certain circumstances, the others raise the concern that the probe could adversely affect neuronal function. We therefore assayed the key AIS-dependent function of action potential initiation, via targeted whole-cell patch-clamp recordings in YFP-Na_V_II–III-transfected vs. untransfected DGCs (Figure 8A). With the synapsin-YFP-Na_V_II–III construct expressed under our experimental conditions, we found no difference in any parameter related to single or multiple spike firing (Figure 8; Table 3). In addition, we profited from the presence of accurate live AIS label in our recorded neurons to investigate structure-function relationships between AIS length and spike characteristics in cultured DGCs. In this dataset we replicated a previous observation obtained with recordings in control-treated, extNF-labeled neurons (Evans et al., 2015): a negative relationship between AIS length and single-spike voltage threshold (Figure 8D, right panel). We also found a significant negative correlation between YFP-Na_V_II–III-labeled AIS length and single spike current threshold (Figure 8C, right panel), as well as a significant positive correlation between AIS length and AP height (Figure 8E, right panel). Finally, in multiple-spiking responses we observed a positive correlation between AIS length and maximum spike number, but in this relatively small sample this effect fell short of statistical significance (Figures 8G,H, left panel; Table 3).

**Table 3:** **Physiological parameters of control or YFP-Na_V_II–III expressing dentate granule cells (DGCs) recorded under baseline conditions**.

Parameter	Mean ± SEM (*n*)		Group comparison	YFP-Na_V_II–III AIS length correlation
	Control	YFP-Na_V_II–III		
*R*_s_ (MΩ)	16.42 ± 0.59 (17)	14.63 ± 0.82 (18)	*t* = 1.77, *p* = 0.09	N/A
*R*_m_ (MΩ)	1300 ± 385.4 (17)	711.1 ± 87.75 (18)	*U* = 110, *p* = 0.16	Sr = 0.43, *p* = 0.07
*C*_m_ (pF)	33.37 ± 1.89 (17)	41.63 ± 2.57 (18)	*t*** = 2.56**, *p* = 0.02	Pr = −0.07, *p* = 0.79
Ithresh (pA/pF)	3.99 ± 0.31 (16)	3.98 ± 0.25 (14)	*U* = 104.0, *p* = 0.76	**Sr = −0.75, *p* = 0.003**
Vthresh (mV)	−25.95 ± 1.54 (16)	−27.83 ± 1.33 (14)	*U* = 90.00, *p* = 0.37	**Sr = −0.61, *p* = 0.02**
Vmax (mV)	31.32 ± 2.65 (16)	36.94 ± 1.22 (14)	*t* = 1.84, *p* = 0.08	Pr = 0.32, *p* = 0.26
AP height (mV)	57.27 ± 3.68 (16)	64.77 ± 2.07 (14)	*t* = 1.71, *p* = 0.1	**Pr = 0.56, *p* = 0.04**
AP width (ms)	1.84 ± 0.18 (16)	1.46 ± 0.07 (14)	*U* = 72.50, *p* = 0.1	Sr = −0.06, *p* = 0.84
Max dV/dt (V/s)	105 ± 18.39 (16)	134.2 ± 12.39 (14)	*t* = 1.28, *p* = 0.21	Pr = 0.28, *p* = 0.33
Max no of spikes	5.41 ± 0.82 (17)	7.75 ± 1.25 (16)	*U* = 100.0, *p* = 0.2	Sr = 0.34, *p* = 0.19

*Group comparisons show results of Mann-Whitney U test or unpaired t-test, and correlations report Spearman’s r (Sr) or Pearson’s r (Pr), for non-parametric and parametric datasets, respectively. Bold text highlights tests where *p* < 0.05*.

## Discussion

**Figure 9:**
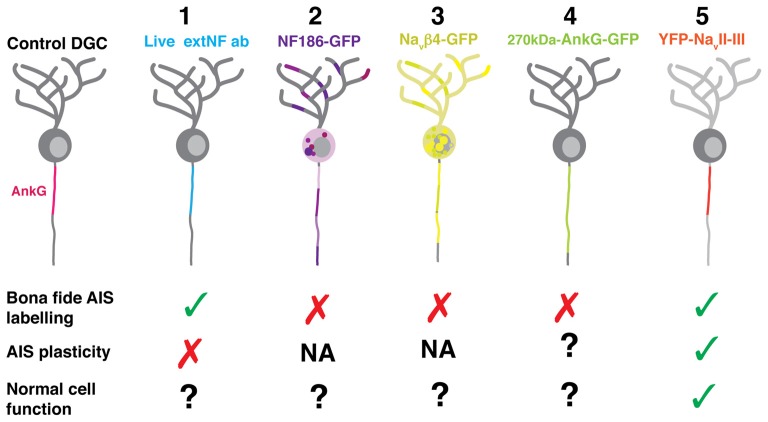
**Diagram summarizing the expression pattern of all five live *in vitro* AIS markers tested. (1)** The live extNF antibody localized at the presumptive AIS endogenous site; however, it did not recapitulate the plasticity phenotype. The same was true for a version of the label conjugated with a 488 nm secondary antibody. **(2)** The NF186-GFP construct did not localize specifically to the AIS. **(3)** The 270kDa-Na_V_β4-GFP construct, in both the full length or the C-terminal tail deletion versions, did not localize specifically to the AIS. **(4)** The 270kDa-AnkG-GFP construct did become localized specifically; however, it artificially lengthened the AIS. **(5)** The YFP-Na_V_II–III fusion construct was localized specifically at the AIS, displayed a shortening phenotype following a 6 h depolarization treatment, and was also shown not to alter neuronal excitability.

To enable live imaging of AIS structure, we assessed five different labeling approaches. Of these, the YFP-Na_V_II–III construct was clearly the most suitable—it accurately revealed both baseline and activity-altered AIS structure, and did not affect intrinsic neuronal excitability (Figure 9). This probe may therefore prove useful in future studies investigating alterations in AIS structure in individual neurons over time.

### Live Label with the extNF Antibody Does Not Accurately Track Structural Plasticity at the AIS

There are significant advantages associated with an immunocytochemical approach to live AIS label in cell culture, including comprehensive coverage across all neurons in a given preparation, labeling that is rapidly effective after minutes, and protocols that do not require disruption of neuronal membrane integrity. Indeed, we found that live application of the extNF antibody is a reasonable, though far from perfect, indicator of baseline AIS length (Figures 2, 3), and it remains entirely suitable for use in conjunction with electrophysiology (Evans et al., 2015) or other acute protocols where it is used to reveal AIS structure live at one single snapshot in time.

However, extNF label does not accurately follow AIS plasticity. After 3 h depolarization its distribution did not shorten at all, and after 6 h it not only underestimated the degree of shortening but also correlated very poorly with AnkG staining (Figure 3). Here our data contrast somewhat with a previous study that found local calcium uncaging at the AIS to produce a 50% reduction in live-labeled extNF fluorescence within 30 min (Schafer et al., 2009). However this calpain-dependent response to injury requires higher levels of calcium entry (Schafer et al., 2009; see also Friedrich, 2004), whereas the calcineurin-dependent AIS plasticity studied here can potentially be activated by much lower levels of calcium influx (Stemmer and Klee, 1994; Graupner and Brunel, 2007; Forbes et al., 2012). It may be, therefore, that the more dramatic process of protease-driven AIS degradation can be more readily reported by the live extNF probe.

Nevertheless, it is surprising that live extNF label does not accurately report hours-scale plastic AIS alterations, given that neurofascin-186 binds directly to AnkG (Davis and Bennett, 1994; Garver et al., 1997; Boiko et al., 2007) and might be expected to follow its distribution faithfully. The molecular features and mechanisms of neurofascin dynamics at the AIS remain entirely unknown, but a prime candidate to explain extNF’s inability to track rapid structural AIS plasticity is cross-linking at the primary antibody level. An effect of cross-linking at the secondary antibody level was ruled out by our experiment showing a lack of live-labeled AIS shortening with monovalently tagged extNF (Figure 3). We can speculate, though, that primary antibody-level extracellular cross-linking of neurofascin molecules may make them resistant to internalization and/or promote de-coupling from the intracellular AnkG scaffold. It is possible, though not trivial, to produce monovalent *F*_ab_-fragment extNF primary antibodies that would not cross-link neurofascin in this way (Bruce et al., 2016) and this may yet prove a fruitful avenue for future studies.

### Unsuccessful AIS Label with NF186-GFP or Na_V_β4-GFP

Our initial attempts to live-label the AIS with genetically-encoded probes were unsuccessful, with neither NF186-GFP nor Na_V_β4-GFP localizing specifically to the proximal axon. These approaches may have suffered from common problems associated with overexpressing sizeable fluorophore fusion proteins, including issues with cellular trafficking or alterations in molecular interactions. The location of fused GFP at the C-terminal tail of neurofascin-186, for example, may have disrupted its FIGQY AnkG-binding motif and prevented successful AIS targeting (Dzhasiashvili et al., 2007). In the case of Na_V_β4-GFP, we cannot account for our inability to replicate previous reports of AIS localization with these same probes (Buffington and Rasband, 2013). We can only speculate that notorious variability in lipofection efficiency, and the associated large difference in transfection reagent exposure times (5–10 min here; 4 h in Buffington and Rasband, 2013) may have contributed to the different results we obtained. It is also worth noting that in the previous publication, the Na_V_β4-GFP signal was amplified via immunocytochemistry with antibodies against GFP (Buffington and Rasband, 2013). It is possible that we have missed potential cells that have a low and specific Na_V_β4-GFP signal that cannot be picked up live, prior to antibody staining. Extensive optimization of these protocols for individual experimental settings might allow successful AIS label with these probes in future studies.

### Overexpression of 270kDa-AnkG-GFP Produces AIS Elongation in Mature Neurons

We found that an alternative, genetically-encoded live AIS reporter—270kDa-AnkG-GFP—was well localized to the AIS, but elongated the structure by ~40%. A similar effect of this construct has been reported by two previous studies in dissociated hippocampal neurons, although both under somewhat different conditions. When over-expressed from the time of plating and throughout the initial stages of AIS development, 270kDa-AnkG-GFP was found to significantly elongate the structure (Galiano et al., 2012). This supported a model of the AIS specification in which the distal AIS boundary is established by the relative timing of ankyrin-B (AnkB) vs. AnkG expression. In this developmental scheme, AnkB is normally expressed early and located in the distal axon, with AnkG later “filling back” the proximal axonal region that is not yet AnkB-occupied (Galiano et al., 2012). Our data show that starting the overexpression of 270kDa-AnkG-GFP at 7 DIV, well past the time of initial AIS formation *in vitro* (Boiko et al., 2003; Yang et al., 2007), can also significantly elongate the structure (Figure 4). They suggest that if the distal AnkG-AnkB boundary still operates after the AIS has initially formed, this boundary is rather flexible.

However, the elongating effects of 270kDa-AnkG-GFP overexpression may be more due to the specific isoform utilized than to overexpression *per se*. When expressed from 3 DIV in cells where all endogenous AnkG expression had been knocked out, 270kDa-AnkG-GFP still produced longer AISs compared to control WT neurons (Jenkins et al., 2015). This effect was not observed with a GFP-fused version of the longer 480 kDa-AnkG isoform, which produced AISs of normal length when expressed from 3 DIV in AnkG-null neurons (Jenkins et al., 2015). It remains to be seen whether this fluorophore-tagged “giant” AnkG is benign when overexpressed in WT conditions, alongside endogenous AnkG production. However, given the importance of this 480 kDa AnkG isoform in AIS formation (Jenkins et al., 2015), overexpressing the 480 kDa-AnkG-GFP construct, or generating transgenic animals that constitutively express 480 kDa-AnkG-GFP (ideally from the endogenous AnkG locus) might be a very promising AIS live-label approach for future investigations.

### YFP-Na_V_II–III is a Suitable Tool for Following AIS Plasticity Live

The most appropriate of our five candidate live-labeling approaches was overexpression of YFP-Na_V_II–III, a construct containing a combination of AIS-localization mechanisms employed by native Na_V_ channels via this intracellular motif (Garrido et al., 2003; Lemaillet et al., 2003; Fache et al., 2004; Bréchet et al., 2008; Gasser et al., 2012). YFP-Na_V_II–III provided accurate label of AIS length under baseline conditions and also after activity-dependent shortening. The genetically-encoded nature of this probe means it may also prove suitable for use in *in vivo* settings.

However, the axonal distribution of this probe was slower to change than those of endogenous proteins. Whilst native AIS components including AnkG and Na_V_ can shorten their distributions after just 3 h of elevated activity (Evans et al., 2015), the YFP-Na_V_II–III distribution took longer to shorten (Figure 7). This is extremely puzzling, given that the Na_V_II–III loop binds directly to AnkG, and is not membrane bound—it should be free to disperse with AnkG, or in unbound form, once the scaffolding molecule is removed from the AIS. We can only speculate that, once localized via an AnkG-dependent interaction, YFP-Na_V_II–III might also have as-yet unidentified associations with more stable non-AIS-specific axonal proteins, or other AIS components that do not shorten (e.g., the microtubule associated protein labeled by the “pIκBα” antibody, Evans et al., 2015).

At first glance, it is also puzzling that overexpression of YFP-Na_V_II–III does not affect neuronal excitability (Figure 8). The construct uses the same mechanism as Na_V_ channels for AnkG binding and AIS localization, so, under the assumption that Na_V_ binding sites on AnkG are normally saturated in DGCs at 10–12 DIV, it might be expected to at least partially out-compete native channels. The resulting reduction in AIS Na_V_ density would then be predicted, on the basis of previous experimental and theoretical work, to impact on action potential initiation (Khaliq and Raman, 2006; Laezza et al., 2007; Kole et al., 2008; Kress et al., 2010; Tapia et al., 2013; Del Puerto et al., 2015). A reduction in whole-cell Na_V_ current was indeed reported with overexpression of GFP-Na_V_II–III in hippocampal neurons of similar maturational status (Garrido et al., 2003), although that study did not assess the construct’s impact on spike firing. In contrast, using Na_V_II–III to localize channelrhodopsin-2 to the AIS did not significantly reduce either whole-cell Na_V_ current or excitability of single or multiple spiking (Grubb and Burrone, 2010b). These differences may be reconciled by cross-study variations in transfection efficiency and therefore expression level: stronger Na_V_II–III overexpression would be more likely to out-compete native channel localization, and while localization of YFP-Na_V_II–III was excellent here, label intensity was certainly on the weaker side. Additionally, it may be possible that AnkG binding sites for Na_V_ are not fully saturated in DGCs at this stage in their maturation, and/or that Na_V_II–III-based constructs do not only out-compete native Na_V_ channels. K_V_7 channels also rely on a similar motif for AnkG binding (Pan et al., 2006; Rasmussen et al., 2007) and act in DGCs to dampen excitability (Martinello et al., 2015). Displacing a mixed population of native Na_V_ and K_V_7 channels might therefore result in balanced effects on action potential initiation.

Overall, we find that synapsin-driven YFP-Na_V_II–III has all the attributes required of a probe for following live structural change at the AIS, at least over longer (>6 h) timescales. When appropriately expressed, it localizes accurately to the AIS without altering baseline length or neuronal excitability, and it can reliably track plastic alterations in the structure. We envisage it being employed in future work to investigate activity-dependent and/or pathological AIS alterations in individual neurons, both *in vitro* and *in vivo*. However, we urge investigators using this probe to take inter-preparation variability into account, and to start by fully characterizing the suitability of label obtained with YFP-Na_V_II–III in their model system of choice.

## Author Contributions

ASD performed all experiments and analysis. MDE produced the synapsin-driven YFP-Na_V_II–III and 270kDa-AnkG-GFP constructs. All authors designed experiments and discussed results. ASD and MSG wrote the article.

## Funding

This research was supported by a Wellcome Trust Research Career Development Fellowship (088301) to MSG, and Medical Research Council 4-year PhD studentships to ASD and MDE.

## Conflict of Interest Statement

The authors declare that the research was conducted in the absence of any commercial or financial relationships that could be construed as a potential conflict of interest.
